# Non-muscle myosin II and the plasticity of 3D cell migration

**DOI:** 10.3389/fcell.2022.1047256

**Published:** 2022-11-10

**Authors:** James M. Cowan, Jacob J. Duggan, Breanne R. Hewitt, Ryan J. Petrie

**Affiliations:** Department of Biology, Drexel University, Philadelphia, PA, United States

**Keywords:** matrix, myosin, motility, cytoskeleton, mechanotranduction

## Abstract

Confined cells migrating through 3D environments are also constrained by the laws of physics, meaning for every action there must be an equal and opposite reaction for cells to achieve motion. Fascinatingly, there are several distinct molecular mechanisms that cells can use to move, and this is reflected in the diverse ways non-muscle myosin II (NMII) can generate the mechanical forces necessary to sustain 3D cell migration. This review summarizes the unique modes of 3D migration, as well as how NMII activity is regulated and localized within each of these different modes. In addition, we highlight tropomyosins and septins as two protein families that likely have more secrets to reveal about how NMII activity is governed during 3D cell migration. Together, this information suggests that investigating the mechanisms controlling NMII activity will be helpful in understanding how a single cell transitions between distinct modes of 3D migration in response to the physical environment.

## Introduction

The ability of a single cell to move through three-dimensional (3D) environments is critical for multicellular life, whether it is during the early stages of development, immune cell surveillance of tissues and organs, or wound healing in an adult organism. The chemical and physical diversity of the 3D environments that cells must navigate is mirrored by the array of mechanisms that single cells can use to achieve motion (Yamada and Sixt, 2019). Determining how motile cells sense and respond to their physical environment will be essential to fully understand the molecular basis of 3D cell migration.

Adhesive cells migrating through 3D extracellular matrices (ECMs) are constantly modifying the surrounding environment (Infante et al., 2018; DeLeon-Pennell et al., 2020) and their own behavior (Friedl and Wolf, 2010) in response to the structure of the matrix through the process of dynamic reciprocity (Xu et al., 2009). This bidirectional interaction between cells and their physical microenvironment is often initiated by mechanosensing, which then triggers mechanotransduction to adjust the molecular mechanisms that single cells use to move [for a recent example see (Pittman et al., 2022)]. As a result of these cell-matrix interactions, a single cell can reprogram its intracellular molecular landscape to achieve motion despite the chemical and physical complexity of the environment it is moving through (Petrie and Yamada, 2016). This process is known as migratory plasticity. Determining the molecular mechanisms responsible for migratory plasticity will be critical to therapeutically control cell movement to improve human health outcomes, such as by promoting fibroblast migration to heal chronic wounds or arresting the movement of malignant cells to slow metatstatic disease.

One approach to determine if a cell is using a unique mechanism or mode of 3D migration is to examine how the cell is generating the necessary force to achieve forward motion. Though the actin-binding motor protein myosin was first isolated from muscle at the time of the American Civil War (Hartman and Spudich, 2012), how it generates force to help cells move through 3D matrices remains a focus of intense investigation (Agarwal and Zaidel-Bar, 2019). The essential details of myosin II function are well known. Myosin II is a filament forming, actin-binding motor protein with ATPase activity that helps to slide actin filaments (F-actin) past each other to generate mechanical force in muscles (Spudich, 1989; Szent-Gyorgyi, 2004). Non-muscle myosin II (NMII) and actin are also amongst the most abundant proteins in migrating cells and have long been considered important components of the molecular machinery driving adhesive cell migration across 2D surfaces (Badley et al., 1980; Herman et al., 1981). However, it is now clear that while NMII activity is dispensable for 2D cell motility (De Lozanne and Spudich, 1987; Even-Ram et al., 2007), it is required for the movement of many cell types through the narrow openings within 3D matrices (Doyle et al., 2012; Petrie et al., 2014).

This review will describe the known modes of 3D cell migration and outline the factors that trigger migratory plasticity to transition cells from one mode to another. We will then characterize how the relocalization of NMII activity in response to the physical environment is essential to reprogram how intracellular forces are generated and applied to the cellular microenvironment to sustain 3D cell motility. Finally, we will highlight how two families of actin binding proteins, tropomyosins and septins, govern NMII localization and activity in migrating cells and other contexts. We speculate that investigating these pathways could provide further insight into the molecular regulation of migratory plasticity. Ultimately, this review will propose that understanding the plasticity of NMII localization and function will provide greater insight into the molecular basis of migratory plasticity in cells responding to their physical environment.

## Migratory plasticity and 3D cell motility

### Movement across 2D surfaces

**FIGURE 1:**
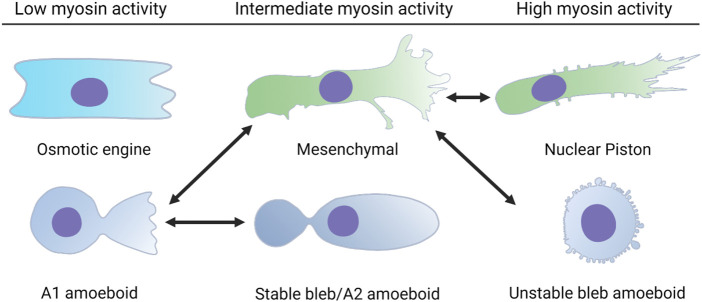
Single cells can switch between distinct modes of 3D cell movement. Each of these modes of migration have different requirements for cell-matrix adhesion and actomyosin contractility. Cells using the osmotic engine or stable bleb modes of movement rely on friction against the surfaces that confine them to achieve forward movement. Cells using the unstable bleb amoeboid, 3D mesenchymal (lamellipodia-based), or nuclear piston (lobopodia-based) modes rely on cell-matrix adhesions to propel themselves forward. The double headed arrows indicate the known transitions that a single cell can use to switch between different modes of movement in response to the physical structure of the ECM and changes to intracellular signaling.

At the end of the 20th century, it seemed like the major questions surrounding how cells migrate had been addressed by investigating adherent cell migration across 2D surfaces. The pathways responsible for adhesion, protrusion, cell body translocation, and contraction of the trailing edge had all been identified and a comprehensive model was proposed to explain how these pathways and components were spatially and temporally coordinated to achieve directional cell movement through the 2D cell motility cycle (Ridley et al., 2003). First, integrin and growth factor signaling at the presumptive leading edge of a crawling cell helps to establish and sustain front-back polarity. Specifically, the small GTPase Cdc42 and members of the PAR complex specify the leading edge through microtubule stabilization (Etienne-Manneville and Hall, 2001). This directs the polarized secretion of new membrane and protein components to the leading edge to bolster further protrusion and adhesion (Schmoranzer et al., 2003). Concurrently with polarized secretion, phosphoinositide 3-kinase (PI3K) and the Rac1 GTPase act through the actin nucleator Arp2/3 to form the dendritic F-actin network within the lamellipodium (Pankov et al., 2005; Suraneni et al., 2012) and push the leading edge forward in combination with local membrane detachment from the underlying cytoskeleton (Welf et al., 2020). As the membrane protrudes forward, nascent integrin-based adhesions are formed. Adhesion maturation then occurs through mechanotransduction using the rigidity sensing machinery (Prager-Khoutorsky et al., 2011), where basal NMII-mediated contractility probes the resistance of the underlying substrate. On rigid surfaces, this probing leads to a positive feedback loop where increased contractility triggers recruitment of integrin and integrin-binding proteins to the adhesion to form additional connections between the integrins and the contractile actomyosin filaments. These connections then begin to exert significant traction stresses against the underlying ECM. A second pool of actomyosin contractility at the rear of the cell is regulated by the small GTPase RhoA, where it governs NMII contractility to sustain retraction of the trailing edge and the disassembly of old cell-matrix adhesions. Importantly, the mechanisms of 2D lamellipodia-based migration remains a vibrant area of research (Welf et al., 2020; Isomursu et al., 2022; Kage et al., 2022; Pasapera et al., 2022; Pittman et al., 2022). However, we now know that lamellipodia-based migration is only one of several modes of migration that can be used by cells moving through 3D environments (Table 1). These modes of migration can be distinguished by whether they are pathfinding or path-generating, as well as their requirement for cell-matrix adhesion, NMII-mediated actomyosin contractility, and matrix metalloproteinase (MMP) activity (Petrie and Yamada, 2016). Further, an individual cell can transition between subsets of these migration modes in response to changes in the chemical and physical microenvironment (Figure 1). Critically, the plasticity of cell migration mechanisms is often mirrored in the plasticity of NMII localization and function.

**TABLE 1:** Modes of single cell migration.

Migration mode	Classification	Protrusion type	MMP required?	NMII required?	Cell-matrix adhesion required?
2D lamellipodia	Not applicable	Lamellipodia	No	No	Yes
Nuclear piston	Path-generating	Lobopodia	No	Yes	Yes
Mesenchymal	Path-generating	Lamellipodia	Yes	Yes	Yes
Unstable bleb amoeboid	Pathfinding	Small, dynamic blebs	No	Yes	Yes
Stable bleb amoeboid (A1)	Pathfinding	Large, stable blebs	No	Yes	No
Rapid amoeboid (A2)	Pathfinding	Small lamellipodia?	No	No	No
Osmotic engine	Pathfinding	Not determined	Not determined	No	No

### Modes of 3D cell migration

When cells are moving through 3D ECM, there are a large variety of physical characteristics they can respond to, ranging from matrix composition and pore size to rigidity and elastic behavior (Doyle et al., 2013). Many studies rely on the presence of fetal bovine serum in the media to trigger cell motility without specifically defining the ligand-receptor pair(s) responsible for initiating the requisite intracellular signaling. It will be essential to completely define these pathways in the future to better understand the molecular origins of cell behavior in response to their physical environment. In the case of primary human fibroblasts, the presence of platelet derived growth factor in the media in the absence of FBS is sufficient to not only trigger cell movement, but also the specific mode of migration in response to the 3D environment (Petrie et al., 2012). We speculate that the pathways governing migratory plasticity will at a minimum require the integration of growth factor signaling and mechanotransduction downstream of integrin-based adhesion. If the pore-size or channel dimensions are large enough, then cells can use the pathfinding modes of migration. However, in matrices with small pore sizes (Wolf et al., 2013) or that are heavily crosslinked (Petrie et al., 2012), cells rely on migration modes that are path-generating, such as MMP-dependent movement through 3D collagen (Infante et al., 2018). Whether pathfinding or path-generating, the central problem that cells must solve to move through these structurally complex 3D environments is how to translocate their large, bulky nucleus through the narrow openings within fibrillar protein matrices (Harada et al., 2014). Interestingly, the nucleus is also required for mesenchymal cells to move in 3D matrices, but dispensable for movement across 2D surfaces (Graham et al., 2018). Though we will introduce each migration mode individually, cells can rapidly transition between modes, transitioning from lobopodia to lamellipodia (mesenchymal), or lamellipodia (mesenchymal) to unstable bleb amoeboid, for example (Figure 1). Importantly, filopodia are another type of protrusion commonly found on cells migrating on 2D surfaces and in 3D matrices, where they help sense the physical and chemical environment to aid in directional migration (Caswell and Zech, 2018; Peuhu et al., 2022). We speculate that cells moving through 3D environments in the body can continuously adjust the mode of their migration in response to the physical environment they are moving through, analogous to a motor vehicle that can switch from two-wheel to four-wheel drive depending on the type of terrain encountered (Talkenberger et al., 2017). Additionally, it remains to be determined how much overlap exists between these different modes of 3D cell motility. For example, the directional flow of water from front to back may be a required for all modes of 3D migration and not just the osmotic engine mechanism that is described below (Stroka et al., 2014). Determining which mechanisms are potentially shared by these distinct modes of migration, along with specifying which cell types are capable of utilizing each mode will clarify the molecular regulation of migratory plasticity (Table 2).

**TABLE 2:** Cell types and signaling pathways associated with each mode of single cell migration.

Migration mode	Examples of cell types capable of using each migration mode	Essential signaling pathways and proteins
Pulled nuclear piston	Primary human mesenchymal cells, fibrosarcoma cells	RhoA-ROCK-NMII, Tpm 1.6, plectin, vimentin
Pushed nuclear piston	Mesenchymal stem cells	TRPV4, Piezo1, nesprin 2
Mesenchymal	Primary human mesenchymal cells, single tumor cells	NEDD9, DOCK3, Rac1, Arp2/3, NMII, MMP
Unstable bleb amoeboid	Single tumor cells, T-cells	FilGAP, ARHGAP22, RhoA-ROCK-NMII, JAK1, STAT3, ezrin, moesin
Stable bleb amoeboid (A2)	Single tumor cells, immune cells	Erk, NMII, Eps8, filamin-A, fascin-1, cofilin-1
Rapid amoeboid (A1)	Normal fibroblasts, normal epithelial cells	Not determined
Osmotic engine	Single tumor cells	RhoA, TRPV4, NHE-1, SWELL-1

### The osmotic engine

One of the most dramatic departures from the molecular mechanisms driving 2D lamellipodia-based motility is the osmotic engine mechanism of 3D migration. Tumor cells using the osmotic engine to migrate along matrix-coated channels completely occlude the channel, meaning all the cellular surfaces are in contact with the channel walls (Stroka et al., 2014; Li and Sun, 2018). When the osmotic engine is active, the movement of the cell body is propelled by the directional flow of ions and water across the plasma membrane at the leading edge and out of the cell at the trailing edge. To achieve directional water flow, the Na^+^/H^+^ exchanger NHE-1 and the aquaporin water channel AQP5 are concentrated in the membrane at the leading edge of the cell (Stroka et al., 2014), while the SWELL-1 chloride channel is enriched in the plasma membrane at the cell rear (Zhang et al., 2022). Interestingly, NHE-1 and SWELL-1 polarization are regulated by Cdc42 and RhoA, respectively. As Na^+^ ions are pumped into the cytoplasm at the leading edge, osmotic pressure drives water influx through AQP5. This initiates the directional flow of water from the front to the back of the cell where it exits the cytoplasm through AQP4, with chloride ion passing through SWELL-1, to propel the cell forward. Interestingly, the osmotic engine can operate in the presence of the actin depolymerizing drug latrunculin A, as well as the NMII inhibitor blebbistatin, indicating that the osmotic engine does not directly rely on actin polymerization or actomyosin contractility. Further, strong adhesion is also unlikely to play a role since adhesion maturation and strengthening rely on NMII-generated contractility (Guilluy et al., 2011).

Given the osmotic engine mechanism does not require NMII activity or actin polymerization, it suggests that directional water flow is prevalent in cells using amoeboid modes of 3D migration, since these can also less dependent on actin polymerization and NMII activity (Paluch et al., 2016). However, it is not clear if the osmotic engine represents a distinct mode of 3D motility, or if directional water flow is a characteristic of all polarized cells migrating through 3D environments. Further study will be required to determine how universal this captivating mechanism may be. Despite the potential ambiguity in which modes of migration may rely on the directional flow of water, there is evidence that cells can transition between water-driven (osmotic engine) and actin polymerization-driven (mesenchymal/3D lamellipodia) cell migration mechanisms by changing NMII localization and activity (Li and Sun, 2018; Zhao et al., 2021). Cells can sense and respond to the magnitude of the hydraulic resistance or pressure they are moving against in tight channels (Prentice-Mott et al., 2013). When hydraulic resistance in front of the moving cell is low, cells rely on an amoeboid mode of migration that does not require actin polymerization to advance the leading edge (Zhao et al., 2021). When hydraulic resistance increases, cells maintain the amoeboid type of migration as there is sufficient NMII-generated contractility to power forward protrusion in the face of this hydraulic pressure. If NMII is not sufficiently enriched at the leading edge, these cells switch to a mesenchymal mode of confined migration that is dependent on Arp2/3 activity.

### Stable bleb amoeboid

**FIGURE 2:**
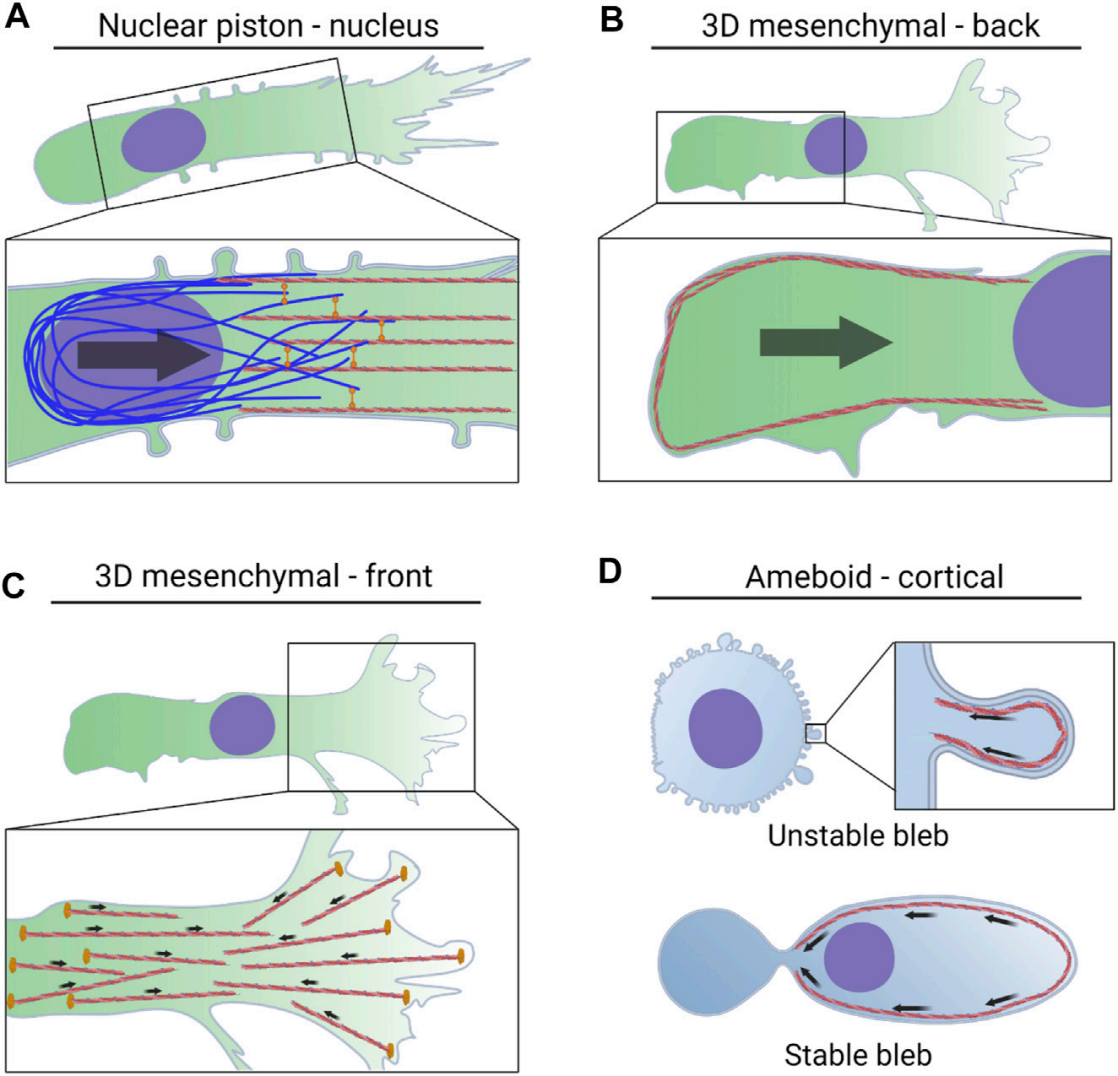
NMII activity powers multiple modes of 3D cell migration via distinct mechanisms. **(A)**. Actomyosin contractility in the front of the cell is attached to vimentin intermediate filaments by the plectin crosslinker. The force generated by this machinery is transmitted to the nucleus via nesprin 3 to help pull the nucleus through 3D matrices. The forward movement of the nucleus increases cytoplasmic pressure to form lobopodial protrusions. **(B)**. NMII activity at the trailing edge is regulated by mechanical stress on the nucleus, tension within the plasma membrane, and the small GTPase RhoA. This actomyosin contractility increases pressure at the rear of the cell and drives water into the nucleus which can lead to blebbing and rupture of the nuclear envelope. **(C)**. NMII activity in leading protrusions can transmit traction forces to the surrounding ECM. This helps to align matrix fibers immediately in front of the cell to promote directional movement. **(D)**. Actomyosin contractility in the cortex increases pressure in amoeboid cells forming small, unstable blebs. NMII-driven retrograde flow propels cells forming stable blebs forward through preexisting channels.

Cells that are unable to adhere to the surrounding environment through integrin-based adhesion are unable to migrate across a 2D surface (Bergert et al., 2015). However, non-adhesive cells can begin to migrate after they are compressed between at least two surfaces. This confinement allows amoeboid cells to generate a large stable bleb and migrate using the friction created by retrograde flow cortical actin and membrane components to propel the cell forward (Liu et al., 2015; Logue et al., 2015; Ruprecht et al., 2015). This mode of migration is used by a wide variety of cells, including transformed epithelial and mesenchymal cells, along with Jurkat T-cells, HL-60 neutrophils, mouse bone marrow-derived dendritic cells, and human monocytes (Table 2) (Liu et al., 2015). The intracellular organization of cells using stable-bleb based migration is dramatically different from cells using lamellipodia-based migration on a 2D surface (Adams et al., 2021). These cells form two large cytoplasmic compartments, separated by a narrow cytoplasmic neck and the nucleus can be found in either the forward or rearward compartment. The organelles and endomembrane system are concentrated in the rear compartment and within the narrow neck separating the forward and rearward compartments. The cortical F-actin network assembles at the tip of the leading bleb, facilitated by the actin-bundling and capping protein Eps8, to increase tension in the cortex and cytoplasmic pressure within the bleb (Logue et al., 2015). Critically, actomyosin contractility drives the retrograde flow of the cortical cytoskeleton that is critical for this low-adhesion mode of cell movement (Hawkins et al., 2011). Cell-matrix adhesion is dispensable for stable bleb migration, and this suggests that cells using this mode of cell migration find existing paths, rather than generating a new path through the 3D ECM. In addition to the retrograde flow beneath the plasma membrane, there is significant cortical contractility at the rear of the cell that is required for the rapid forward movement of these cells in confinement (Venturini et al., 2020). Importantly, this pool of cortical NMII activity at the rear of the cell also contributes to the movement of adherent mesenchymal cells using 3D lamellipodia-based migration (Lomakin et al., 2020) and we speculate it may be a universal feature of cells migrating through 3D matrices (Figure 2).

Interestingly, when clusters of cells are confined within non-adhesive channels, they can switch their mode of migration from a traction force-based migration mechanism (Friedl et al., 2012) to an adhesion-independent amoeboid mode (Pages et al., 2022). This collective amoeboid movement requires RhoA activity and concentrated actomyosin contractility at plasma membranes of cells at the rear of the cluster where it helps to steer the cell cluster. However, unlike stable bleb amoeboid migration, there is no significant retrograde flow associated with the forward movement of the cluster.

It is not clear how cells transition from adhesion-based mechanisms such as 3D mesenchymal migration to non-adhesive mechanisms such as the stable bleb mode. It has been proposed that early in development, before the production of abundant ECM, adhesion-independent motility could be essential for cells to move and position themselves within developing tissues (Venturini et al., 2020). Thus, we speculate that conditions of physical confinement allow cells with sufficient cortical retrograde flow and negligible integrin expression to migrate along preexisting paths within tissues (Paluch et al., 2016). However, boosting cell-matrix adhesion by increasing integrin expression could transition the cells to the adhesion-based, 3D mesenchymal mode of migration that utilizes lamellipodia based protrusions and matrix remodeling to generate their paths through fibrillar 3D matrices such as type I collagen.

### 3D mesenchymal migration

Cells that form robust cell-matrix adhesions can migrate through dense fibrillar matrices using lamellipodia-based protrusions and traction forces generated by actomyosin contractility within stress fibers near the front of the cell (Petrie et al., 2012; Wang et al., 2019; Doyle et al., 2021). This anterior contractility is coordinated with cortical contractility at the rear of the cell to help disassemble cell-matrix adhesions and keep the trailing edge advancing in synch with protrusion at the leading edge. In addition, this mode of migration requires the secretion of MT1-MMP to cleave collagen fibers within the ECM to facilitate the passage of the large, bulky nucleus (Infante et al., 2018). These cells use polarized signaling at the leading edge that is classically associated with lamellipodia movement across 2D surfaces (Petrie et al., 2012). Specifically, Cdc42 and Rac1 activity, along with the PI3K-generated second messenger phosphatidylinositol (3,4,5)-trisphosphate (PIP3), are polarized towards the leading edge and enriched at the ends of long pseudopodial protrusions in small lamellipodia. At the very front of the cells, rearward directed traction forces generated by NMII help align and stiffen matrix fibers to direct the continuous forward movement of the cell (Doyle et al., 2021). On synthetic matrices, this ability to increase tension within the matrix, coupled with asymmetric adhesion, has revealed a dramatic slingshot effect in some cases. Tension within the matrix can be combined with the rapid release of cell-matrix adhesions to dramatically catapult the cell body forward (Wang et al., 2019). NMII-mediated contractility within the cortical cytoskeleton along the sides of the cells acts to prevent the formation of protrusions off the axis of migration (Fischer et al., 2009; Elliott et al., 2015). Actomyosin contractility within the cortical cytoskeleton at the rear of the cell assists in pushing the nucleus forward from the back (Hetmanski et al., 2019; Lomakin et al., 2020). Interestingly, the coordination between the pools of NMII-generated contractility at the front and back of the cell may help to steer the cell through the 3D matrix (Godeau et al., 2022). It will be fascinating to learn how these mechanisms interface with more localized steering mechanisms like protrusion stability (Petrie et al., 2009) or cortical control of protrusion formation (Fischer et al., 2009; Elliott et al., 2015) to achieve directional migration such as during 3D chemotaxis. Since the presence of the nucleus is required for 3D cell migration (Graham et al., 2018), it is also possible that the numerous connections between the nucleus and cytoskeleton are required to help organize the distribution and application of forces all over the cell to achieve productive cell movement (Wang et al., 2009).

Interestingly, 3D mesenchymal cells can transition between at least three different modes of migration depending on the following factors: integrin-based adhesion, actomyosin contractility, and the amount of cross-linking in the ECM (Petrie and Yamada, 2015). If their integrin-based adhesions are downregulated, along with reduced actomyosin contractility, these cells adopt a rapid, amoeboid form of migration characterized by a round cell body and single protrusion tipped by a small lamellipodia (Liu et al., 2015). If actomyosin contractility is elevated while cell-matrix adhesion remains low, such as when MMP activity is inhibited in tumor cells (Wolf et al., 2003), they transition to a round migration mode associated with small unstable blebs. Finally, if mesenchymal cells encounter a covalently cross-linked matrix, or if MMP activity is reduced so they cannot rely on proteases to remodel the matrix, they polarize their actomyosin contractility in front of the nucleus (Petrie et al., 2014; Petrie et al., 2017). This anterior actomyosin contractility is connected to the nucleus and is anchored in cell-matrix adhesions at the front of the cell to help pull the nucleus forward (see nuclear piston mechanism below).

### Unstable bleb amoeboid

Rounded cells using unstable bleb-based amoeboid migration mechanisms result from an increase in cortical actomyosin contractility, coupled with low adhesion to the surrounding ECM. Unlike amoeboid migration using stable blebs, unstable bleb-based migration still requires some degree of cell-matrix adhesion (Wolf et al., 2003; Ferrari et al., 2019; Guzman et al., 2020). This mode of migration is used by a wide variety of transformed cells, as well as T-cells in response to sphingosine-1-phosphate (Robertson et al., 2021). In tumor cells, the increased actomyosin contractility is primarily triggered by reduced MMP activity (Sahai and Marshall, 2003; Wolf et al., 2003), though there are additional regulatory pathways governing this mode of 3D migration (Sanz-Moreno et al., 2011). This elevated contractility, which is potentially a combination of cortical NMII and NMII localized to the back of the cell (Poincloux et al., 2011; Asante-Asamani et al., 2022), triggers a rounding up of the cell, likely due to these cells lacking strong enough cell-matrix adhesion to resist the inward pulling forces generated by NMII. A combination of increased cytoplasmic pressure and reduced attachment of the plasma membrane to the underlying actin cortex (Charras et al., 2005; Charras et al., 2008), causes rapid blebbing at the plasma membrane. These blebs are rapidly retracted by actomyosin contractility in the bleb neck (Taneja and Burnette, 2019). Dynamic blebs are also hotspots for the formation of new cell-matrix adhesions to facilitate the forward movement of these cells (Ferrari et al., 2019). These interactions can be augmented by the physical intercalation of lateral cellular protrusions, such as blebs, into the spaces in the surrounding ECM to help propel the cells forward through a process termed chimneying (Reversat et al., 2020). Interestingly, this mode of migration may be a hybrid of pathfinding and path-generating. Cells using the amoeboid modes of migration typically have more deformable nuclei, allowing easier passage of the nucleus through small openings within the matrix (Harada et al., 2014). However, additional evidence suggests that rounded amoeboid cells may upregulate other MMPs to compensate for reduced MT1-MMP activity and to degrade and remodel the ECM (Orgaz et al., 2014).

### Nuclear piston mechanisms

The unstable bleb amoeboid mode of migration is not the only mechanism that is triggered by the inhibition of MMP activity. When adhesive mesenchymal cells confront 3D matrices that pose a barrier to the movement of the nucleus, they link nuclear motion to protrusion formation and enlargement to find their way forward (Petrie et al., 2014; Lee et al., 2021). Specifically, cells that maintain robust cell-matrix attachment following the increase in actomyosin contractility that is triggered by MMP inhibition (Petrie et al., 2017) or encountering linearly elastic, crosslinked 3D matrices (Petrie et al., 2012), can use their nuclei like a piston to increase pressure in front of the nucleus. This increase in pressure corresponds with the formation of lobopodial protrusions to generate paths through the matrix when degrading the matrix is not an option (Petrie et al., 2017; Lee et al., 2021). The nuclear piston mode of 3D migration can be subdivided into two distinct mechanisms depending on whether the required actomyosin contractility is pushing the nucleus forward from the back or pulling it forward from the front. Mesenchymal stem cells moving through protease-resistant 3D material, such as viscoelastic alginate hydrogels coupled to the cell adhesion peptide RGD (arginine, glycine, and aspartate), use cortical actomyosin contractility at the back of the cell to push the nucleus forward (Lee et al., 2021). The forward movement of the nucleus increases anterior cytoplasmic pressure, which increases tension on the plasma membrane and triggers an influx of ions through mechanically activated channels. Water then flows into the cell enlarging the cytoplasm in front of the nucleus. This enlarged cellular protrusion acts as a wedge to forge a path through the hydrogel by providing additional space for the nucleus to move into. Alternatively, primary human mesenchymal cells, such as dermal fibroblasts, intestinal myofibroblasts, and de-differentiated chondrocytes, polarize their actomyosin contractility in front of the nucleus to pull the nucleus forward when moving through covalently crosslinked, linearly elastic material, such as cell-derived matrix (CDM). As the nucleus is pulled forward, it can also increase the hydraulic pressure in the cytoplasmic compartment in front of the nucleus (Petrie et al., 2014). This elevated pressure corresponds with a switch from low-pressure lamellipodia to high-pressure lobopodial protrusions (Petrie et al., 2012). It remains to be determined whether the influx of ions and water that are integral to the pushed-piston mechanism (Lee et al., 2021) have a similar role in maintaining cytoplasmic volume when the pulled-piston mechanism is operating (Petrie et al., 2014).

While it is unclear whether mesenchymal stem cells can use more than one mode of 3D cell migration, primary human fibroblasts can switch between at least three distinct modes of cell migration. When moving through linearly elastic 3D matrices, like CDM, they use the RhoA-ROCK-NMII signaling axis to pull the nucleus forward, pressurize the cytoplasm, and turn off the Rac1-Arp2/3 machinery responsible for forming lamellipodial protrusions (Patel et al., 2021). This triggers a switch from actin-driven lamellipodial migration to pressure-based lobopodial protrusions. Inhibiting actomyosin contractility in these environments causes the cells to revert to low-pressure, lamellipodia migration within ∼15 min (Petrie et al., 2014). Further, reducing adhesion in confined channels will switch the migration mode to a rounded, amoeboid form of motility characterized by low actomyosin contractility (Liu et al., 2015). Interestingly, all the above migration modes can be distinguished by the unique localization and function of NMII. This suggests understanding the signaling and mechanotransduction pathways controlling NMII activity and its subcellular localization will be essential to understand how cells transition from one mode to another in response to changes in their physical environment.

## The plasticity of NMII during 3D cell migration

The fundamental details of NMII structure and function have been carefully investigated for many decades (Spudich, 1989; Szent-Gyorgyi, 2004). The heavy chains of NMII are responsible for converting the chemical energy present in ATP to molecular motion *via* a series of conformational changes driven by ATP binding, hydrolysis, and release of the resulting ADP. The small GTPase RhoA is responsible for activating downstream effectors like Rho-dependent kinase (ROCK) which then can phosphorylate the myosin regulatory light chains to increase NMII activity. Despite the wealth of information on the structure and regulation of NMII, the molecular mechanisms that drive the plasticity of NMII localization and function during 3D migration are not fully understood. Interestingly, there are several distinct subcellular regions where this force-generating machinery can be precisely localized and regulated within each of the distinct cell migration mechanisms described above. Further, the localization of NMII activity can be governed by mechanosensing of the extracellular physical environment and subsequent intracellular mechanotransduction. Next, we will describe the most recent findings regarding how NMII is localized in cells using the distinct modes of 3D migration and the signaling pathways regulating its activity at each subcellular location.

### NMII at the trailing edge

The cortical cytoskeleton underlying the plasma membrane at the rear of the cell can be enriched with RhoA and NMII during the migration of adhesive cells across 2D surfaces (Ridley et al., 2003). The actomyosin contractility at the rear of the cell aids in adhesion disassembly and contraction of the trailing edge (Vicente-Manzanares et al., 2007). The reciprocal distribution of Rac1 and RhoA activities at the front and back of the cell, respectively, is at least partially dependent on inhibitory crosstalk between the two pathways (Iden and Collard, 2008). The classical function of NMII activity at the back of the cell is conserved within several modes of 3D cell migration, but it may not strictly rely on RhoA regulation to direct its function (Lomakin et al., 2020; Venturini et al., 2020). However, the spatial control of RhoA activity at the back of the cell can control the direction of cell movement through 3D matrices (Chi et al., 2014).

As described above, the nucleus can be considered as the master regulator of 3D migration. The nucleus is the rate-limiting step of cell movement when moving through the narrow openings of engineered channels or fibrillar protein matrices. Confusingly, the nucleus is also required for 3D cell movement (Graham et al., 2018), but not required for cell migration across 2D surfaces (De Lozanne and Spudich, 1987; Even-Ram et al., 2007). There could be at least two reasons why the nucleus is specifically required for the movement of cells through 3D environments. First, the nucleus is subject to significant mechanical forces during cell migration (Nader et al., 2021), and those forces can be sensed by the nucleus to trigger an increase in contractility at the rear of the cell rear to help push the nucleus forward (Lomakin et al., 2020; Venturini et al., 2020) (Figure 2B). Specifically, the nuclear envelope to acts as a mechanosensor of the physical environment to regulate NMII activity at the trailing edge. The nuclear envelope can accommodate excess membrane in the form of folds or wrinkles across its surface. When the nucleus encounters a narrow opening in the 3D matrix and begins to move through it, the mechanical stress on the nucleus can increase pressure inside the nucleus and tension in the nuclear envelope. This increased tension and pressure can lead to bleb formation and even a transient rupture of the nuclear envelope (Denais et al., 2016). In migrating mesenchymal cells using lamellipodia-type protrusions (Lomakin et al., 2020) and in stable-bleb amoeboid cells in zebrafish (Venturini et al., 2020), mechanical stress on the nucleus triggers the release of calcium from the endoplasmic reticulum and increased tension within the nuclear envelope that leads to the rapid loss of membrane wrinkles. These two factors in turn trigger the recruitment the calcium-dependent phospholipase cPLA2 to the nuclear envelope where it produces the second messenger arachidonic acid. Arachidonic acid increases NMII activity at the rear of the cell by recruiting the regulatory myosin light chain 2 to the cortical cytoskeleton. This increased NMII activity at the trailing edge provides additional pushing forces on the nucleus to force it past barriers in the surrounding 3D environment. Further work is required to determine if this is a universal feature of all modes of 3D migration using contractility, or if it is restricted to cells using 3D lamellipodia-based migration.

An additional mechanism regulates NMII contractility at the back of the cell during 3D mesenchymal cell migration. Excess plasma membrane can accumulate at the rear of the cell (O'Neill et al., 2018), leading to a reduction in plasma membrane tension at the rear of cell relative the plasma membrane in front of the nucleus (Hetmanski et al., 2019). This lower tension at the rear of the cell corresponds with an enrichment of plasma membrane invaginations known as caveolae. Caveolae and their resident proteins caveolin-1 and cavin-1 have long been known to act as signaling hotspots (Zajchowski and Robbins, 2002), and only more recently has their role as mechanosensors of membrane tension been revealed. When plasma membrane tension is low, the caveolae recruit the RhoA guanine exchange factor Ect2 to the rear of the cell where it activates RhoA (Hetmanski et al., 2019). The active RhoA binds and activates ROCK and PKN2 to increase actomyosin contractility at the trailing edge to facilitate the translocation of the nucleus and cell body through 3D matrices. How this mechanism is related to the nuclear mechanosensor and the cPLA2 pathway controlling NMII at the rear of the cell is unclear at this time. However, the increased RhoA activity at the rear of the cell can also increase pressure inside the nucleus (Mistriotis et al., 2019), which could then trigger activation of cPLA2 and further increase NMII activity at the rear of the cell. Specifically, actomyosin contractility can increase cytoplasmic pressure (Patel et al., 2021). Thus, the RhoA activity at the rear of the cell likely increases cytoplasmic pressure and drives water into the nucleus to increase nuclear pressure (Mistriotis et al., 2019) and aid in the translocation of the nucleus through narrow openings (Keys et al., 2022). This elevation in nuclear pressure increases membrane tension and promotes blebbing and rupture of the nuclear envelope. Though it has not been directly demonstrated, it is possible that the cPLA2-and RhoA-dependent mechanisms controlling NMII activity at the back of the cell could represent a positive feedback loop where RhoA activity at the rear of the cell promotes further contractility *via* the pressurization of the nucleus and subsequent activation of NMII at the plasma membrane due to the production of arachidonic acid (Figure 2).

### NMII within the cortical cytoskeleton

The NMII activity that is localized to the cortical F-actin network immediately beneath the plasma membrane in front of the trailing edge has several important roles in cells migrating through 3D environments. NMII activity can accelerate the retrograde flow of the F-actin network underneath the plasma membrane, along with associated proteins and lipids (Shih and Yamada, 2010). This accelerated retrograde flow creates sufficient friction to propel the cell forward through predetermined channels (Bergert et al., 2015) (Figure 2D). In addition, the modes of migration defined by the presence of small, unstable blebs rely on cortical NMII activity to retract the expanding bleb and prevent it from becoming oversized (Taneja and Burnette, 2019). Contractility in the cortical actomyosin network can also increase tension in the plasma membrane to open mechanically-gated channels and increase ion and water flow into the cell to increase cytoplasmic pressure and control cell volume (Tao and Sun, 2015; McEvoy et al., 2020; Lee et al., 2021). Finally, the cortical NMII activity that increases membrane tension helps to prevent the formation of lateral protrusions and promote directional cell movement, but also acts as a point of regulation to locally reduce membrane tension (Fischer et al., 2009; Elliott et al., 2015). These local changes in pressure and membrane tension may help initiate protrusion formation to help steer the direction of 3D cell motility in cells using 3D mesenchymal migration mechanisms.

Important questions to address are how is NMII integrated with the cortical F-actin network and what controls its activity there? A hotspot of cortical actomyosin assembly is close to the leading edge (Anderson et al., 2008). This is where F-actin is polymerized and anchored to the plasma membrane *via* proteins like ezrin (Robertson et al., 2021). Multiple NMII isoforms (NMIIA and B), along with other myosin family members like myosin 18A, can co-assemble with cortical stress fibers and F-actin at the leading edge (Beach et al., 2014; Billington et al., 2015; Shutova et al., 2017). Subsequent retrograde flow of this network can lead to sorting of NMIIA and B, with NMIIA remaining more peripheral and NMIIB concentrated closer to the cell body. This distribution of NMIIA and B is consistent with the finding that NMIIA is important for generating traction forces in cellular protrusions (Shutova et al., 2017; Doyle et al., 2021), while NMIIB is critical for helping to move the nucleus through 3D matrices (Thomas et al., 2015), as well as directing adhesion formation beneath the cell body (Doyle et al., 2021). Though these findings are derived mainly from examining stress fibers embedded within the cortical F-actin network, we speculate similar mechanisms could be operating to concentrate NMII within the cortical F-actin network itself. Importantly, cortical F-actin can also assemble at the sides of the cell away from the leading edge in response to the actin-nucleating activities of Arp2/3 and mDia1 (Bovellan et al., 2014). The architecture of the resulting cortical F-actin network is governed by the balance between branched F-actin and long F-actin filaments nucleated by Arp2/3 and mDia1, respectively. This mechanism is a way to control membrane tension independently of NMII activity. Interestingly, inhibiting Arp2/3 increases NMII intercalation into cortical F-actin and also increases membrane tension (Truong et al., 2021), consistent with the finding that Arp2/3 inhibition increases cytoplasmic pressure dependent on NMII activity (Patel et al., 2021). Once the cortical actomyosin network is assembled, the associated NMII activity can be controlled by at least two distinct mechanisms. Calcium entry through the plasma membrane can feedback on NMII activity in the cortex to further increase contractility (Chen et al., 2013). Secondly, the small GTPase RhoB can be translocated to the plasma membrane from the endomembrane system with the help of the kinesin KIF13A (Gong et al., 2018). Once at the plasma membrane, RhoB activates NMII within the cortical cytoskeleton to increase cytoplasmic pressure and trigger the small dynamic blebs that are characteristic of unstable bleb-based amoeboid motility.

### NMII near the leading edge

Cell-matrix adhesion is critical for the path-generating modes of 3D migration, such as during lamellipodia- (Doyle et al., 2012) and lobopodia-based (Petrie et al., 2012) motility (Figure 2A,C). The adhesions that form at the very front of the cell are strengthened over time by actomyosin contractility with cycles of membrane protrusion and retraction helping to apply traction forces to the matrix fibers to promote directional cell migration (Wang et al., 2019; Doyle et al., 2021). NMIIA is the NMII isoform responsible for generating these traction forces at the leading edge of the cell in 3D. The initial engagement of integrins with the matrix triggers the further recruitment of the focal adhesion proteins vinculin and paxillin to form stable adhesions that are strongly adherent to the matrix and associated with negligible retrograde flow. Thus, the contractility that drives adhesion maturation also increases the local matrix alignment at the front of the cell. NMIIA activity at the leading edge could also be regulated by local influxes of calcium through the TRPM7 stretch-activated Ca2^+^ channels in a feedback loop initiated by NMII-generated tension within the plasma membrane (Wei et al., 2009). Alternatively, the collagen adhesion-mechanoreceptor DDR1 is an important component of machinery responsible for generating traction forces and remodeling the collagen matrix. DDR1 associates with the myosin phosphatase Rho-interacting protein (MRIP) which regulates the clustering and growth of DDR1 containing adhesions by increasing NMII activity at the leading edge of the cell (Coelho et al., 2020). Finally, the local RhoA activity at the leading edge could also govern actomyosin contractility at the front of the cell (Machacek et al., 2009).

**FIGURE 3:**
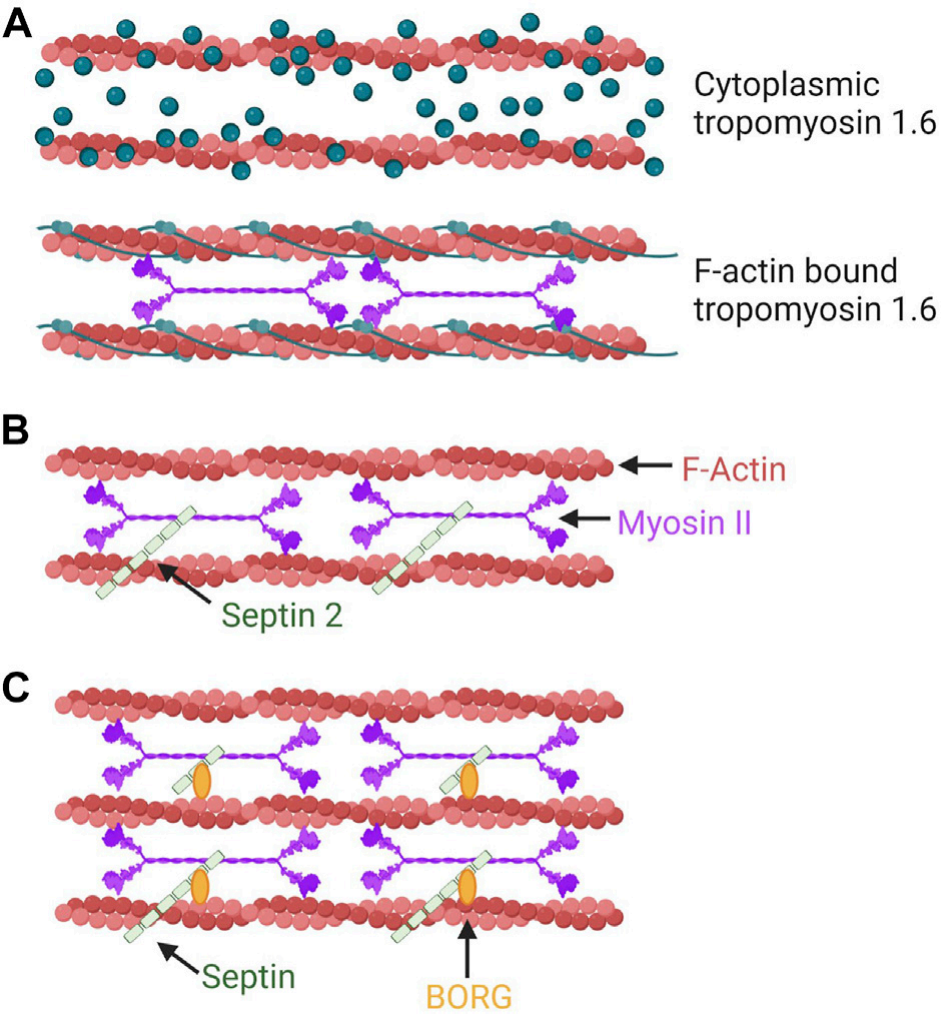
Tropomyosins, septins, and the BORG proteins can modulate the NMII activity within discrete populations of actin filaments. **(A)** Tpms can modulate the activity of NMII on F-actin. In this example, the association of Tpm 1.6 with actin stress fibers increases NMII activity. This mechanism aids in pressure-based 3D migration mechanisms such as the nuclear piston. **(B)** SEPT2 can promote actomyosin contractility through a direct interaction with NMII on F-actin. **(C)** BORG (binder of Rho GTPases) proteins are effectors of the small GTPase Cdc42 that bind and regulate septins to modulate intracellular force production by NMII.

When primary human mesenchymal cells encounter linearly elastic, covalently crosslinked 3D matrices they activate a second pool of NMII contractility that also acts in front of the nucleus. The function of this perinuclear pool of NMII is to pull the nucleus forward through the tight spaces in the 3D ECM (Petrie et al., 2014; Thomas et al., 2015). We speculate that NMIIB is the isoform responsible for generating and maintaining the tension necessary to pull the nucleus forward. A critical question is how is this cytoplasmic contractility applied to the nucleus to pull it forward? Interestingly, it is not necessarily a direct connection. Vimentin intermediate filaments are required for the nucleus to be pulled forward by actomyosin contractility through 3D CDM (Figure 2A) (Petrie et al., 2017). The cytoskeleton crosslinking protein plectin connects the cytoplasmic F-actin network in front of the nucleus to the basket of vimentin intermediate filaments that surround the nucleus in response to substrate rigidity (Marks et al., 2022). Specifically, this network is assembled in response to soft substrates and requires NMII activity, consistent with mechanosensing by the classical rigidity-sensing machinery. Once this nuclear piston pulling machinery is connected, it becomes activated *via* an unknown mechanism that can sense the elastic behavior of the surrounding matrix (Petrie et al., 2012; Petrie et al., 2014). The actomyosin filaments that are responsible for pulling the nucleus forward to generate pressure are further distinguished from actomyosin filaments that create traction forces by the presence of the acting-binding protein Tpm 1.6 (Figure 3A) [(Sao et al., 2019); and see Tropomyosins below]. The association of Tpm 1.6 promotes NMII activation to generate the force necessary to pull the nucleus forward, while Tpm 2.1 negatively regulates the actomyosin machinery responsible for traction forces and matrix remodeling. Consistent with traction forces and pressure being critical for nuclear piston migration, both Tpm 1.6 and 2.1 are required for 3D cell migration. In addition to nesprin 3, nesprin 2G has been implicated in contributing to 3D cell movement by coupling the nucleus directly to the cytoplasmic F-actin network. These connections transmit tension to the nucleus (Arsenovic et al., 2016) and are concentrated in the front of the nucleus in cells migrating through a confined environment (Davidson et al., 2020), consistent with actomyosin contractility acting through nesprin 2G to help pull of the nucleus forward. Further work is needed to fully understand how the distinct cytoskeleton-nucleoskeleton connections are coordinated to achieve 3D migration.

## Additional mechanisms that could control local recruitment and activation of NMII during 3D cell migration

Given that the plasticity of 3D cell migration is a relatively new field of study (Friedl and Wolf, 2010), many additional questions remain regarding how NMII activity is localized and controlled when cells switch between distinct migration modes. It is likely, therefore, that better understanding how the localization of NMII and the mechanotransduction triggering its activation will be essential to understand how cells are able to navigate the diverse physical matrix structures present in the body. Septins and tropomyosins are components of the cytoskeleton that have already been shown to contribute to some of the mechanisms driving cell motility through 3D ECMs. The following provides some additional information about how these highly adaptable proteins are known to influence NMII localization and activity which may also play a role in directing NMII plasticity in migrating cells.

### Tropomyosins

Tropomyosins (Tpms) are a large family of filament forming, F-actin binding proteins that can specify the function of the F-actin filaments with which they associate (Figure 3A) (Gunning et al., 2015). The diversity in F-actin filament function can then be explained by the forty unique Tpm mRNAs that are expressed from four genes (Geeves et al., 2015). An example of how Tpm association with actin can change depending on the location and function of the F-actin filament comes from examining the distribution of Tpm isoforms in cells adhering to a 2D surface (Tojkander et al., 2011). In these cells, Tpm 1.6 associates along the full length of dorsal stress fibers, while Tpm 2.1, Tpm 3.1, and Tpm 3.2 are enriched at the ends of these dorsal stress fibers where they correspond to vinculin-positive focal adhesions. An early example of their role in cell migration and protrusion formation comes from the discovery that the expression of Tpm 3.12 triggered the loss of lamellipodia and lamellipodia-independent 2D migration (Gupton et al., 2005). Further, Tpm 1.6 is enriched in actin stress fibers across the full length of cells migrating over 2D surfaces but redistributed exclusively in front of the nucleus in cells using the nuclear piston to migrate through 3D matrices (Sao et al., 2019).

Tpms can co-assemble with polymerizing actin fibers whose formation is nucleated by formins (Johnson et al., 2014), though evidence exists that Tpms can also be recruited to pre-assembled F-actin networks (Appaduray et al., 2016). *In vitro* studies have revealed that the association of different Tpm isoforms with actomyosin filaments can change the characteristics of NMII function within those filaments. These characteristics include sliding filament velocity driven by NMII activity, the amount of time the NMII is continuously walking along the filaments (processivity), and duty load as an indirect measure of the force generated by the filaments (Gateva et al., 2017). In the presence of other actin-binding proteins it gets a little more complicated. Tpm can compete with other actin-binding proteins to change the architecture and function of the F-actin network, along with NMII activity (Hu et al., 2019). Essentially, increased disorder in the F-actin network leads to higher tension and traction forces generated by NMII activity in response to increased Tpm 3.1/3.2 driving α-actinin out of the network. Further, Tpm 3.1/3.2 are not uniformly distributed along the length of F-actin, instead they are associated with NMII head domains, rather than the long NMII heavy chain tails or with α-actinin binding sites (Meiring et al., 2019). Tpm 3.1 expression is required for NMII association with those F-actin networks. Tpm 1.6 and Tpm 2.1 also localize to distinct domains within dorsal stress fibers (Sao et al., 2019). Importantly, unique functions are associated with these spatially segregated Tpms. Tpm 1.6 increases actomyosin contractility to generate increased cytoplasmic hydraulic pressure, while Tpm 2.1 negatively regulates the actomyosin contractility responsible for generating traction forces. Post-translational modifications in the form of N-terminal acetylation may be a critical regulator of Tpm function and help to diversify the functional characteristics of NMII associated with distinct subsets of F-actin (Reindl et al., 2022).

### Septins, anillin, and the Borg

Septins are filament forming proteins that regulate the organization and activity of contractile actomyosin structures required for normal migration and tumor cell invasion by directly binding non-muscle myosin II, scaffolding myosin binding partners, and facilitating the assembly of higher order structures (Figure 3B). [For a comprehensive review of septins, see (Spiliotis and Nakos, 2021)]. Which septin family members are involved and their specific roles can vary by cell type, dimensionality of the ECM, and mode of migration. *In vitro* experiments revealed that septin 2 (SEPT2) binds directly to a coiled-coil region of NMIIA (Figure 3B) (Joo et al., 2007). SEPT2-NMIIA binding is required to maintain intact actomyosin stress fibers at the center of the cell and, during cell division, maintain the degree of myosin light chain phosphorylation necessary for completing cytokinesis. SEPT2 may indeed scaffold the kinases ROCK and CRIK to activate NMII, as ROCK and CRIK associate whether or not NMII is present, and the interaction of NMIIA with SEPT2 is required for MLC di-phosphorylation to complete cytokinesis (Joo et al., 2007). In mouse podocytes, active NMIIA forms a complex with SEPT7 and the t-SNARE protein SNAP3 (Wasik et al., 2017). Here, SEPT7 stabilizes the formation of a complex between NMIIA and SNAP23 but suppresses NMIIA activity. In immature rat hippocampal neurons, a wreath-like network of septin filaments containing SEPT7 acts as a scaffold to localize NMIIB to the base of F-actin bundles within incipient neurites to regulate somatic contractility and protrusion formation (Radler et al., 2022). In human renal epithelial cells migrating using a mesenchymal mode, septin filaments comprised of septins 2/6/7/9 localize to contractile transverse arc stress fibers where SEPT9 crosslinks actin bundles, while depletion of SEPT2 does not alter levels of activated NMIIA (Dolat et al., 2014). Similarly, in amoeboid mouse lymphocytes, knocking down SEPT7 does not change NMII expression or phosphorylation of NMIIA heavy and light chains. However, SEPT7 in lymphocytes (Tooley et al., 2009; Gilden et al., 2012) and SEPT9 in melanoma cells (Farrugia et al., 2020) do help modulate cortical contractility.

Anillin is a scaffold protein that was first discovered for its role in governing actomyosin contractility at the cytokinetic ring during the final stages of cell division (Field and Alberts, 1995). More recently, anillin has been found to coordinate with septin filaments within the cytokinetic ring during ring assembly and cytokinesis. Specifically, anillin-like proteins in yeast help to organize a double ring of septin filaments on either side of the central ring of actomyosin (Chen et al., 2020; Arbizzani et al., 2022). Interestingly, anillin has also been found to regulate actomyosin filaments in interphase epithelial cells, where it recruits vinculin to the medial-apical actomyosin network at the surface of epithelial cells (Arnold et al., 2019). This results in an increase in tension in this network that may be partially dependent on septins.

Borg (binder of Rho GTPases) proteins, also known as Cdc42EPs, are Cdc42 effectors that bind and regulate septins. Cdc42EP3 (Borg2), -EP5 (Borg3) and -EP1 (Borg5) have been studied with regard to their potential impact on NMII function. Cdc42EP3 directly binds SEPT2 and SEPT7 (Joberty et al., 1999; Joberty et al., 2001) and F-actin, and these interactions generate filamentous septin and actin networks in cancer-associated fibroblasts (CAFs) (Figure 3C) (Calvo et al., 2015). Depleting Cdc42EP3 or SEPT2 reduces actomyosin stress fibers and active NMII *in vitro* and reduces the ability of CAFs to remodel matrix *in vitro* and *in vivo*. In 3D collagen, Cdc42EP5 predominately localizes with active contractile actomyosin at the cell cortex in melanoma cells (Farrugia et al., 2020). Cdc42EP5 depletion, but not depletion of any other Borg proteins, reduces the phosphorylation of myosin light chain 2 levels without affecting MLC2 expression. Still in 3D collagen, Cdc42EP5 interaction with SEPT9 increases NMII activity and is required for actomyosin-dependent melanoma invasion. On 2D surfaces, filamentous Cdc42EP1, which interacts with SEPT7 (Joberty et al., 2001), co-aligns with SEPT7, actin stress fibers, and active NMII above the nucleus in myocardial endothelial cells (Liu et al., 2014). Loss of Cdc42EP1 or SEPT7 disrupts the tripartite association of septin, Cdc42EP1, and actomyosin filaments. Further, there is a decrease in perinuclear NMII activity and a reduction of persistent directional migration in 2D and 3D environments, though the relationship remains correlative between Cdc42 EP1 expression, disrupted structures and migration (Liu et al., 2014).

## Conclusion

Overall, we have learned much in the nearly 160 years since myosin was first isolated about how this essential actin-binding motor protein converts chemical energy to mechanical energy to achieve motion at the cellular, tissue and organismal scales. The utility of actomyosin contractility is also exemplified by the numerous ways that non-muscle cells have adapted this force-generating system to achieve migration through 3D matrices. It remains an open question as to how many ways there are for cells to migrate through the physically diverse environments within the body. By continuing to investigate the unique roles of NMII in producing the physical forces to achieve cell motility, we predict the rules governing mode switching will be revealed.
